# Anticoagulation Therapies and microRNAs in Heart Failure

**DOI:** 10.3390/biom15101411

**Published:** 2025-10-03

**Authors:** Lucia Spartano, Maria Lombardi, Chiara Foglieni

**Affiliations:** Cardiovascular Research Center, IRCCS San Raffaele Hospital, 20132 Milan, Italy; spartano.lucia@hsr.it (L.S.); lombardi.maria@hsr.it (M.L.)

**Keywords:** Heart Failure (HF), anticoagulation drugs, Clopidogrel, Aspirin, Warfarin, Apixaban, Rivaroxaban, Edoxaban, Dabigatran, Digoxin, Ivabradine, microRNAs (miRs)

## Abstract

Heart Failure (HF) remains a major cause of mortality despite the advances in pharmacological treatment. Anticoagulation therapies, including Clopidogrel, Aspirin, Warfarin, and novel oral anticoagulants (NOACs) such as Apixaban, Rivaroxaban, Edoxaban, and Dabigatran, are frequently administered to HF patients to prevent thromboembolism and adverse, life-threatening outcomes (e.g., stroke and myocardial infarction). In these settings, drug resistance and variability in responsivity to therapeutic approaches are challenging issues. Recent studies suggest that non-coding RNAs, particularly microRNAs (miRs) may play a modulatory role in HF therapy context, affecting drug efficacy. Specific miRs have been associated with resistance to Clopidogrel (e.g., miR-223 and miR-26a), Aspirin (e.g., miR-19b-1-5p and miR-92a) and Warfarin (e.g., miR-133 and miR-137). Moreover, Digoxin, a cardiac glycoside acting also over bleeding risk, upregulates miR-132, which is involved in HF-associated cardiac alteration and hypertrophy. Evidence linking miR expression to NOAC pharmacodynamics, cardiac remodeling and regulation of the coagulation is growing. These findings highlight the need of deeply harnessing the potential of miRs as predictive biomarkers or therapeutic targets in HF. Improving the knowledge on the relationship between miR and anticoagulant drugs in HF patients will contribute to personalization of the anticoagulant therapies, aimed at enhancing patient responsivity and minimizing adverse effects, ultimately improving patient life quality.

## 1. Introduction

Heart Failure (HF) may be induced by any condition involving the alteration of the structure or function of the left ventricle, and its incidence has progressively risen in the last decade along with an increase in the associated comorbidities and of long-term mortality for cardiovascular causes [1,2,3]. An optimized guideline-directed medical treatment (GDMT) is not always setup in patients upon diagnosis. In fact, despite the global progress in the treatment strategies, significant therapeutic gaps persist in HF and are critical in patients with comorbidities (e.g., pulmonary arterial hypertension), leading to increased risk of worsening of the cardiovascular signs and symptoms, with frequent hospitalizations [4,5,6].

Following both AHA [7,8] and European Guidelines [9], the outpatient therapies frequently include anticoagulation and/or antiaggregant drugs both in patients with concomitant HF and atrial fibrillation (AF) and in those with HF at risk of stroke or of thromboembolic complications (according to the Virchow triad component alteration) [10].

Antiaggregant medications are mostly represented by P2Y12 or cyclooxygenase inhibitors (e.g., Clopidogrel and Aspirin) and are prescribed as single or double antiplatelet therapy (DAPT). The increased risk of major bleeding events and of negative outcomes due to drug resistance following anticoagulant administration in HF sometimes overcomes the advantages of this strategy or implicates fine-tuned administration. In these settings, a deep knowledge of the mechanism of action of the anticoagulants and of other coagulation-related drugs is relevant for the purpose of optimizing more personalized, efficient therapies in HF.

Anticoagulant medications may act at different levels of the coagulation cascade and are represented by vitamin K inhibitors (e.g., Warfarin), factor Xa inhibitors (e.g., Apixaban, Rivaroxaban, Edoxaban), and direct thrombin inhibitors (e.g., Dabigatran) [5,11,12].

Although not belonging to the class of anticoagulants, it is worth considering the two other medications frequently co-prescribed with anticoagulants. The first one is Digoxin, a glycoside known from the 18th century for its regulatory properties over cardiac contractility and risk of bleeding [13,14,15]. The second one is Ivabradine, a benzazepine sino-atrial node current inhibitor preserving hemodynamic stability in patients without interfering with responsivity to antiaggregant therapy [16].

In the setting of a continuously increasing rate of HF, along with life expectancy elongation [5,11,12], further improving the knowledge on how drugs interact at molecular levels with the human organism is pivotal to improve the therapy personalization aimed at primary prevention, or at reducing failures due to drug resistance or comorbidities development.

In this context, why focusing on microRNAs is a legitimate question. The non-coding RNAs demonstrated a potential initially reconducted reconducted to fetal gene reprogramming [17], then progressively extended to diagnosis and therapy [18,19,20,21,22]. A wide literature is currently available on PubMed about microRNAs (miRs) in the heart > 10,000 publications (including in vitro studies), and of them > 2400 are mentioning HF. Basically, the miRs are single strand small RNAs modulating target genes or proteins. The miR targets may include drug targets, and miRs also demonstrated the capability to functionally interact directly with medications, thus contributing to therapeutic efficacy/resistance. Acting on miRs that could interfere with anticoagulant drugs may prospectively support the design of more personalized therapies for HF, and identification of the miRs related to anticoagulants in HF is emerging [23,24].

Here, we provide information about the functional relationships among several main coagulation-related medications for the treatment of HF and non-coding RNAs, specifically the circulating miRs.

## 2. Antiplatelet Drugs

### 2.1. Clopidogrel and Ticagrelor

Clopidogrel, also known by the commercial name Plavix, was first approved by the United States Food and Drug Administration (FDA) in 1997 and by the European Medicines Agency (EMA) in 1998 (Table 1).

Clopidogrel is an active thienopyridine derivative, specifically a prodrug of the active thiol metabolite activated via a two-step reaction involving the enzyme encoded by the hepatic cytochrome P450 2C19 (CYP2C19) gene [25]. Clopidogrel irreversibly and selectively inhibits the binding of adenosine diphosphate (ADP) to platelet P2Y12 receptor and the subsequent activation of the glycoprotein GPIIb/IIIa complex (Table 1).

A 75mg oral dose of clopidogrel is 50% absorbed from the intestine, while 85–90% of an oral dose undergoes first pass metabolism by carboxylesterase 1 in the liver, producing an inactive carboxylic acid metabolite [26,27]. About 2% of clopidogrel is oxidized to 2-oxoclopidogrel [28]. Clopidogrel is excreted 50% in the urine and 46% in the faeces. The remaining, circulating Clopidogrel irreversibly binds to platelets for their lifetime, or approximately 8–11 days [29].

Ticagrelor was approved by EMA in 2010 and by FDA in 2011 with the commercial name Brilique or Brilinta (Table 1), frequently co-administered with Aspirin. At difference from Clopidogrel is a reversible P2Y12 platelet inhibitor and is associated to low mortality outcomes but a higher risk of bleeding with respect to Clopidogrel in relationship with treatment efficacy [30,31,32,33,34].

**Figure 1:**
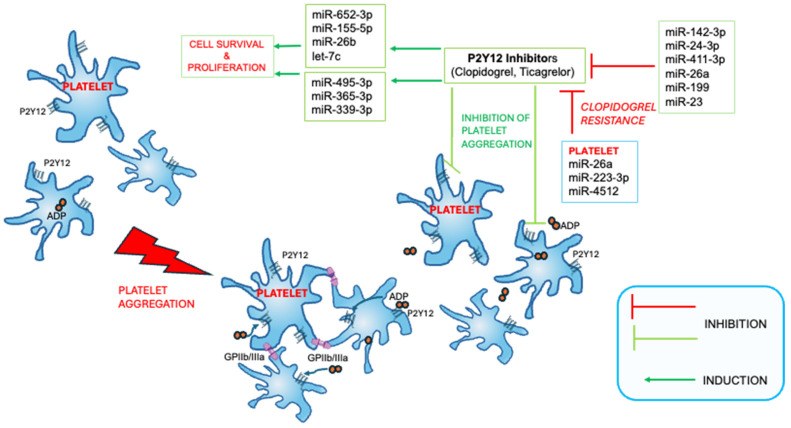
**Schematic representation of the action of Clopidogrel and Ticagrelor on platelets**. The schematization shows P2Y12 inhibitors binding to platelet P2Y12 receptors, hampering that of ADP required for platelet aggregation, and the parallel induction of several miRs involved in cell survival. Other circulating miRs playing an inhibitory action over Clopidogrel and Ticagrelor and contributing to drug resistance are presented.

Several miRs involved in Clopidogrel and Ticagrelor action and resistance have been identified and object of investigation (Figure 1).

The prospective, single-blind, randomized comparative clinical trial TCHIRB-I021003 (NCT02101437) evaluated the correlation of seven miRs (namely miR-96-5p, miR-495-3p, miR-107, miR-223-3p, miR-15a-5, miR-365-3p, and miR-339-3p) with the response to antiplatelet therapy in 155 patients with coronary artery disease (CAD) under DAPT (including combination of Aspirin with either with Clopidogrel or Ticagrelor), scheduled for elective stent implantation [35]. The study reported a strong correlation between the miRNA-365-3p levels in Platelet Rich Plasma and high on-treatment platelet activity, and that the expression of miR-339-3p, miR-365-3p, and miR-495-3p was highest with Clopidogrel vs. Ticagrelor.

The miRs are present in the bloodstream, carried by cells, macrovesicles, or Argonauts, and a clinical trial, the TIGER M Study (NCT02071966), was designed to investigate in vivo the mechanisms of action of Clopidogrel and Ticagrelor as changes in microparticles and miRs. The study enrolled 56 patients (33 males and 23 females) with Non-ST Elevation Acute Coronary Syndrome, randomized to Ticagrelor or Clopidogrel, and preliminary data showed an opposite modulation of miR-652-3p, miR-155-5p, miR-26b, and let-7c associated with antiplatelet therapy (upregulation by Clopidogrel, downregulation by Ticagrelor). Interestingly, one miR modulated by the two drugs, namely the miR-652-3p, was related to the prediction of acute kidney disease in patients with HF [36]. The miR-652-3p is implicated in numerous pathways at molecular level [37], but its specific modulation by P2Y12 inhibitors does not appear to further compromise patient conditions, thus it could be of therapeutic interest. However, although providing findings relevant to acute coronary syndrome, this study included only acute patients, and we cannot exclude that the exacerbation of the impact of antiplatelet drugs on several miRs occurs in acute conditions but not in HF/CAD.

Another study identified the circulating miR-142-3p, miR-24-3p, and miR-411-3p as potential markers for Clopidogrel resistance (determined by the presence of Clopidogrel active metabolites in the bloodstream after 7 days of therapy) in 66 patients with CAD treated with DAPT (Aspirin + Clopidogrel) [38]. The serum miR-26a, miR-199, and miR-23, and the platelet miR-26a, miR-223-3p, and miR-4512 were preliminarily suggested as predictive of Clopidogrel resistance in CAD by other reports [38,39,40]. Unfortunately, no data about the mechanism interrelating Clopidogrel with these miRs were reported. Furthermore, the modulation of miR-24, miR-26, and miR-199 was evaluated in preclinical studies at myocardial level, showing the association with BIM/NOS, BMP/SMAD, and NACC2 signaling, respectively, using modified antisense oligonucleotides (antagomiRs) in models of ischemic disease or angiogenesis [41,42,43]. However, these findings were obtained in the absence of anti-P2Y12 drugs. To preliminarily assess the existence of functional interplay among the miRs potentially related to anti-P2Y12 drugs, in silico analysis of miR-142-3p, miR-24-3p, miR-411-3p, miR-26a, miR-199, and miR-23 networking was performed by miRTargetLink 2.0 [44]. The miR-411-3p failed to show interaction with the others, in turn associated in a network including 11 target genes (namely ACVR1B, CHEK1, E2F2, GSK3B, HMGA1, MAFB, PTGS2, SMAD4, TGFB2, TGFBR1, and JAG1). Using STRING v12 engine [45], we depicted the associated GO biological processes, which were implicated in positive regulation of cardiac epithelial to mesenchymal transition (EMT), cardiac morphogenesis, and endocardial activation (e.g., GO:0062043, GO:0010718, GO:0003208, GO:0060411, GO:0001837, and GO:0048762). Overall, we may surmise that deregulation of the expression levels of the circulating miR-142-3p, miR-24-3p, miR-411-3p, miR-26a, miR-199, and/or miR-23 due to an altered synthesis by the damaged heart in patients with HF, contributes to Clopidogrel inhibition, and ultimately to drug resistance.

The miR-223 is one of the three most abundant platelet miRs and targets the P2Y12 receptor [35,46,47], playing a paracrine function. Low circulating levels may be found in patients poorly responding to Clopidogrel or after antiplatelet therapy [48].

A study on 444 patients with CAD and on DAPT [39] focused on the interplay between long non-coding RNA growth arrest-specific 5 (GAS5) functional polymorphism rs55829688 and platelet miR-223, demonstrating that the polymorphism is involved in Clopidogrel resistance and that GAS5 overexpression reduced miR-223-3p but increased P2Y12 expression in platelets. This is consistent with the proposed role of miR-223 (binding 3′untranslated region (3′UTR) of the P2Y12 mRNA) in the regulation of P2Y12 expression and with the protective role of GAS5 in HF and other cardiac disorders [49,50]. Moreover, in subjects with the *YP2C19* poor metabolizer genotype (i.e., with reduced capability to activate the drug), the GAS5 regulates P2Y12 expression and Clopidogrel response by acting as a competitive endogenous RNA for miR-223-3p, thus suggesting a connection between Clopidogrel activation pathway and platelet responsivity to the drug.

Other polymorphisms and miRs were associated to Clopidogrel resistance and to risk of bleeding (e.g., miR-6076 rs1463411 G polymorphism) during Clopidogrel treatment in acute coronary syndromes (ACS) [38,51,52,53,54,55]. Interestingly, the targets of miR-6076 include *CYP2C19* and *P2RY12*, and the rs1463411 polymorphism is associated with a decrease in the P2RY12 protein on platelets, thus suggesting an inhibitory effect over function and expression level of the Clopidogrel target, which may lead to low responsivity to the therapy.

It is also worth mentioning the involvement of circular RNAs in Clopidogrel responsiveness. Specifically, a microarray study in 50 patients with stable CAD (25 responders and 25 non-responders to Clopidogrel) identified the downregulated hsa_circ_0076837, hsa_circ_0057714 (targeting AOX1), and hsa_circ_0076957 (targeting COL19A1) as candidate biomarkers of Clopidogrel resistance [55]. Preliminary evidence that circ-0076957 regulates COL19A1 through targeting miR-4512 was provided by in vitro validation on liver cells, leading the authors to hypothesize that the modulation of collagen COL19A1 by miR-4512 might affect collagen and thrombus formation, and consequently the Clopidogrel efficacy [55]. A strong emphasis on the need for further mechanistic studies is given in the conclusions of this work.

### 2.2. Aspirin

Aspirin, commercial name of acetylsalicylic acid synthetized in 1897, is a salicylate [56] classified as non-steroidal anti-inflammatory and antiplatelet agent, administered in a variety of conditions, due to its capability of reducing inflammation, pain, and fever, as well as preventing platelet aggregation [57]. Acetylsalicylic acid-based medicinal products were approved in 2016 by EMA and FDA (Table 1).

Aspirin primarily works by inhibiting the enzyme cyclooxygenase (COX), involved in the production of prostaglandins and thromboxane [58,59]. Specifically, Aspirin irreversibly binds to COX-1 (Table 1), preventing it from converting arachidonic acid into prostaglandin H2 (a precursor to thromboxane). This also affects COX-2, though at a slower rate, leading to the production of Aspirin-triggered lipoxins, which can have anti-inflammatory effects. Excretion of salicylates occurs mainly through the kidney. The half-life of Aspirin in the circulation ranges from 13 to 19 min.

**Figure 2:**
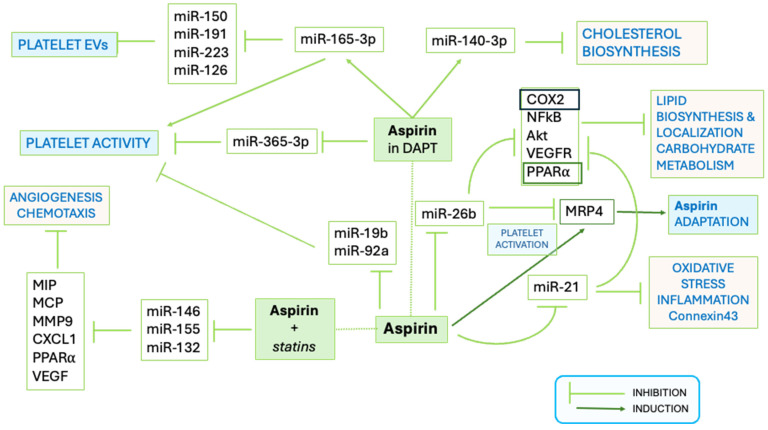
**Schematic representation of the functional effects of interaction of Aspirin with miRs**. The pathways affected by Aspirin alone and in combination with antiplatelet drugs or statins are shown. Platelet activation, release of extracellular vesicles (EVs), but also metabolic processes are targeted by Aspirin (modified from https://doi.org/10.1155/2021/6830560).

The use of Aspirin in HF remains controversial due to dose-associated mortality risk; although low doses are useful for secondary prevention [60], its benefit in primary prevention of HF is not fully supported, and carefulness is required in recommending Aspirin for that purpose [61,62]. How the effects exerted by Aspirin on coagulation and platelet reactivity involve its modulatory action over circulating and platelet miRs (Figure 2) are partially elucidated and still object of studies. The miR-26b, a negative regulator of Multidrug Resistance Protein-4 (MRP4, acting as ATP binding cassette membrane transporter), was downregulated in platelets from patients on chronic Aspirin treatment [63]. In vitro and in vivo studies suggested that Aspirin upregulates MRP4 via PPARα and that megakaryocyte adaptation to Aspirin involves an increase of MRP4 in platelets [64,65]. Interestingly, in silico analysis by miRTargetLink 2.0 [44] shows COX2 and FGF2 genes among the targets of miR-26b (Supplementary Figure S2). The diminished expression of the miR-26b in patients under chronic Aspirin therapy may lead to COX increase and ultimately contribute to Aspirin adaptation. Furthermore, preliminary evidence showed that Aspirin may exert a protective action over endothelial cells from Ox-LDL-associated damage by supporting the transcription of FGF2 [66].

Aspirin indirectly modulates the release of miR-126 from platelets to plasma by inhibiting platelet activation, thus hampering its usefulness as a cardiovascular disease biomarker [67] and reducing its activity associated with thrombin generation [68]. The mesenchymal stem cells in the heart and in the surrounding adipose tissue exposed to Aspirin upregulated the expression of several miRs, including hsa-miR-4734, 10a-5p, hsa-miR-4267, hsa-miR-3197, and hsa-miR-3182, and decreased the production of arachidonic acid metabolites, potentially impacting over metabolism involved in heart repair processes [69].

In patients with CAD taking Aspirin, the expression of circulating miR-483-5p was more elevated (n = 19) than in those not taking the drug (n = 46), and the miR was proposed to target 14 cardiac genes, involved in multiple myocardial pathogenetic mechanisms [70].

Moreover, in patients with CAD and on DAPT with Aspirin and Clopidogrel, the miR-365-3p and miR-223 were proposed as platelet biomarkers [35,46,47]. Aspirin resistance was related to the downregulation of miR-19b-1-5p [71] and of miR-92a [72]. Downregulated miR-19b-1-5p was associated with platelet aggregation on aspirin and a higher risk of major adverse cardio-cerebrovascular events in a cohort of 965 ACS patients in DAPT [73]. Among the targets of miR-19b-1-5p [44] (Supplementary Figure S1), there are the two platelet ADP receptors, P2RY1 and P2RY12- [74,75,76]. Polymorphisms on these receptors (e.g., P2RY1 polymorphism SNPs rs106577, rs701265—also known as 1622A/G mutation, *P2RY12* SNP rs9859538, rs7615865) were associated with different platelet reactivity and inadequate Aspirin responsivity in CAD [77,78].

## 3. Anticoagulant Drugs

### 3.1. Vitamin K Inhibitor: Warfarin

Warfarin is known under the brand names Coumadin and Jantoven (Table 1) and was approved by FDA in 1954. Warfarin was already on the market before the establishment of the European Medicines Agency in 1995, and the relative documents on EMA RDW catalogue dated from 2013.

Warfarin belongs to the class of dicumarols and remains a reference in the field. Warfarin is a competitive inhibitor of the vitamin K epoxide reductase complex subunit 1 (VKORC1), an enzyme essential for activating available vitamin K [79]. Through this mechanism (Table 1), Warfarin decreases the amount of functional vitamin K, reducing the synthesis of active coagulation factors without affecting blood viscosity. Specifically, the reduced form of vitamin K, namely vitamin KH_2_, is a cofactor in the γ-carboxylation of coagulation factors VII, IX, X, and of thrombin, which induces a conformational change allowing the factors to bind Ca^2+^ and phospholipids. Biologically inactive non-carboxylated factors interrupt the coagulation cascade.

Adverse effects such as necrosis, purple toe syndrome, osteoporosis, valve and artery calcification, and drug interactions have been documented with Warfarin use. Carefulness is required for establishing the appropriate Warfarin dose, which should be thoughtfully monitored and adjusted [80,81,82,83,84,85]. In addition to an impairment of the quality of life, HF-associated hospitalization was reported to increase under conditions of sub-optimal Warfarin control [84].

**Figure 3:**
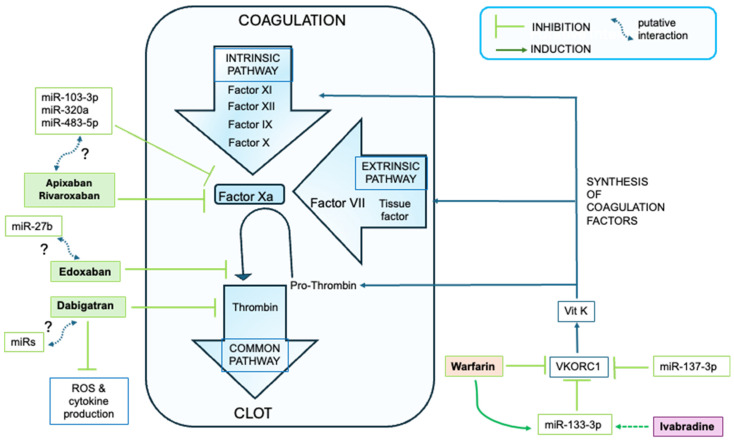
**Functional interplay between coagulation cascade factors and anticoagulant drugs and I_f_ blocker**. The putative (arrows with dotted line) and validated (arrows with continuous line) interaction of miRs involved in the regulation of coagulation pathways with drugs or coagulation factors is shown.

Warfarin exists as a racemic mixture of two isomers, namely R-isomer and S-isomer (quantitatively prevalent). The metabolism of both occurs mainly at the hepatic level, through the mitochondrial cytochrome P450 family 2 subfamily A or C members, namely CYP3A4, CYP1A2, and CYP1A1 for R-isomer, and the CYP2C9 enzyme for S-isomer. Polymorphisms of both CYP1A1 and CYP2A9 were known, but those of CYP1A1 failed to produce significant metabolic effects [86]. So far, associations between presence of CYP2C9 polymorphism in Warfarin-treated patients and bleeding or poor coagulation-related complications have been reported, indicating a potential usefulness of patient genotyping for identifying patients requiring particular care during Warfarin treatment [86,87,88,89]. Polymorphisms in miR precursors, leading to individual variability potentially affecting drug response, have also been found. An example is provided by polymorphism rs2660304 of miR-137, which is suggested to directly target VKORC1 in liver cells and is associated with Warfarin maintenance dose in patients with AF [90] (Figure 3). Interestingly, miR-137 was proposed as a biomarker of unstable angina and of acute ST elevation myocardial infarction in patients [91]. The miR-137 is dependent on Nourin^®^ (an alarmin registered by Nour Heart, Inc. as a blood-based biomarker for the rapid detection of myocardial ischemia before infarction) and is also related to α-1-antichymotrypsin protein, a member of the Serpin family involved in tissue protection, whose levels are more elevated in the myocardium of patients with severe HF supported by a Left Ventricle Assist Device than in healthy individuals [92].

Another interesting case is that of miR-133 variants, one of which, namely the polymorphism rs45547937 in MIR133A2, is affecting the requirement of high doses of Warfarin [93]. The importance of these findings relates to the knowledge that miR-133 is constitutively co-expressed with VKORC1 in human hepatocytes and includes among its targets the VKORC1 gene (Figure 3, Supplementary Figure S3), and through binding in a highly evolutionary-conserved binding site, may affect VKORC1 protein expression [94]. Moreover, the hsa-miR-133b was proposed as a possible reference factor for the Warfarin dosage algorithm in a Chinese Han population [95]. The downregulation of myocardial miR-133a was associated to high NT-proBNP and HF severity in a prospective study on 83 patients with CAD undergoing coronary bypass surgery, but the therapy or coagulative status of patients was not mentioned, thus, no connection between miR-133 and Warfarin was reported by this study [96]. More interestingly, changes in the expression level of miR-133a-3p in circulating cells from patients undergoing mechanical valve replacement and who had received Warfarin for at least 3 months were reported, indicating that Warfarin may affect the expression of miR-133. Overall, these findings suggested an involvement of miR-133 in the regulation of coagulation, acting upstream the synthesis of coagulation Factors [97]. The thyroid hormone (TH), 3-5-3-triiodothyronine (T3), has been successfully applied to a model of cardiac ischemia and reperfusion for recovering myocardial miR-133a and ATP-sensitive mitochondrial potassium channel activity [98] but no analogous studies are currently available in humans with HF.

### 3.2. Oral Anticoagulants

#### 3.2.1. Factor X Inhibitors: Apixaban and Rivaroxaban

Apixaban, a pyrazolopyridine compound, is an oral, direct, and highly selective factor Xa (FXa) inhibitor (Table 1) of both free and bound FXa, as well as of prothrombinase, and is independent of antithrombin III for the prevention and treatment of thromboembolic diseases [99]. Apixaban was approved by EMA in 2011 and by FDA in 2012 and marketed under the name Eliquis (Table 1). 

The 56% of an orally administered Apixaban dose is recovered in the faeces and <30% of the dose in the urine; about 85% of the dose recovered in the urine was the unchanged parent compound [100]. Clinical usefulness of patient genotyping for supporting the optimization of Apixaban dose was suggested by a Japanese report on a cohort of 44 patients with AF treated with Apixaban. The study showed that plasmatic ratio concentration/dose is highest in carriers of ATP-binding cassette subfamily G2 421A/A (ABCG2 421A/A) and CYP3A5*3 polymorphisms, but not in those with polymorphisms of *ABCB1* [101,102].

The administration of oral anticoagulation drugs is indicated in the co-presence of AF and HF because of the associated increased risk of stroke [103], and Apixaban has demonstrated superior effectiveness and safety profile compared to vitamin K antagonists in patients with AF, HF, and low body weight [104,105,106]. Moreover, compared to another frequently prescribed novel oral anticoagulant (NOAC) with different pharmacokinetic properties, namely Rivaroxaban [105], Apixaban leads to a lower incidence of major ischemic or hemorrhagic events in patients with AF and was more effective in preventing stroke [107,108].

Rivaroxaban, a monocarboxylic acid amide, was approved under the commercial name of Xarelto by EMA in 2008 and by FDA in 2011 (Table 1) [109], basically for the prevention and treatment of deep vein thrombosis. In 2025, the generic NOAC version was also approved for reducing the risk of major cardiovascular events in patients with CAD and major thrombotic vascular events in those with peripheral artery disease [108]. Rivaroxaban is scarcely catabolized and excreted by urine and faeces (in the proportion of 2/3 and 1/3, respectively).

The Factor 10 gene, encoding for factor X (i.e., the precursor of factor Xa), showed one validated target miR, namely miR-335-5p, and 16 predicted target miRs by miRTargetLink 2.0 [44]. In addition, the hsa-miR-103-3p emerged from a recent study on COVID-19 as strongly correlated with factor Xa activity [110,111]. Overall, these findings suggest that a relationship between miR and NOACs is plausible.

A Chinese multicenter study (NCT03161496) showed in 257 patients with non-valvular AF in therapy with Rivaroxaban the association of the single nucleotide polymorphism USD3 rs76292544 with 12-month bleeding events and of NCMAP rs4553122, PRF1 rs885821, PRKAG2 rs12703159, rs13224758, and POU2F3 rs2298579 with peak anti-FXa level but also the preventive efficacy of Rivaroxaban pharmacokinetic–pharmacodynamic over thromboembolism in a subset of 136 patients vs. 26 healthy controls [112]. Two miRs, namely miR-320a and miR-483-5p (Supplementary Figure S4, no validated targets were available for miR-320a), involved in cardiac remodeling were positively associated with both anti-Xa activity and pharmacokinetic–pharmacodynamic profiles of Rivaroxaban [113,114,115,116,117] (Figure 3). Notably, the upregulation of miR-483-5p was also related to Aspirin assumption (see also Section 2.2), but no specific studies on the miR influence on patients with HF in Aspirin plus Rivaroxaban therapy are currently available. Furthermore, to the best of our knowledge, no analogous study is available for Apixaban, nor specifically designed for HF. In addition, the only EU prospective randomized clinical trial (EudraCT Number 2015-002755-94) addressed to evaluate the modulation of miRs and target genes involved in coagulation, left ventricle, and atrium function in patients with AF before and after treatment with Apixaban or Warfarin was prematurely closed for unknown reasons; thus, no findings are available.

#### 3.2.2. Thrombin Activation-Inhibitor: Edoxaban

Edoxaban, another monocarboxylic acid amid, called with commercial names of Lixiana, Roteas, or Savaysa (Table 1), is a NOAC approved by both EMA and FDA in 2015.

Edoxaban is a rapidly acting, orally bioavailable, selective competitive inhibitor of the coagulation factor Xa [118] (Figure 3). Specifically, Edoxaban prevents the cleavage of prothrombin into thrombin and amplification of protein factors needed for clottiìng and is indicated to reduce the risk of stroke and systemic embolism [119]. Unchanged Edoxaban is the predominant form in plasma (peak of plasma concentration within 2 h from administration [120], half-life post-oral administration 10–14 h) and is not related to ABCB1 (ATP Binding Cassette Subfamily B Member 1, also called MDR1), SLCOB1 (solute carrier organic anion transporter family member 1B1, encoding for a protein which transports molecules from blood to the liver), CYP2C9, and VKORC1 variants [121]. Edoxaban is eliminated primarily as unchanged drug in urine and is only partially metabolized in the liver by CYP3A4 and carboxylesterase 1 (CES1, targeting hsa-miR-197-3p, a biomarker of cardiac damage [122]) and by the ABCB1 enzymes [121]. The ABCB1 has several target miRs related to coagulation/bleeding (Supplementary Figure S5). It is worth mentioning that the mechanism of post-transcriptional regulation of CYP3A4 is still incompletely understood, despite the existence of in vivo and in vitro indications that miR-27b down-regulated CYP3A4 protein [123,124]. The miR-27b is a repressor of mitochondrial oxidative phosphorylation, involved in HF, myocardial infarction, and cardiac remodeling via fibroblast growth factor 1 [125,126,127,128,129]. Whether there is any functional interrelationship between Edoxaban and miR-27b remains to be established (Figure 3).

#### 3.2.3. Direct Thrombin Inhibitor: Dabigatran Etexilate

Dabigatran etexilate, known with the commercial name of Pradaxa, was approved by EMA in 2008 and by FDA in 2010 (Table 1).

Dabigatran etexilate (ID.: BIBR0951) is a structurally complex oral prodrug based on a benzimidazole core, hydrolyzed to its active form, namely Dabigatran, by carboxylesterases both at intestinal and hepatic levels (i.e., intestinal CES2 and hepatic CES1) [130] and is expelled from the body via renal excretion (half-life of 12–17 h) [131]. Dabigatran is a reversible competitive thrombin inhibitor that directly inhibits the conversion of fibrinogen to fibrin by thrombin (Figure 3), impairing the clotting process and acting as an anticoagulant [132] (Table 1). In addition, Dabigatran inhibits platelet aggregation stimulated by thrombin and von Willebrand factor, but not by other pathways such as ADP- or thromboxane A2-induced aggregation. In addition, Dabigatran does not impair the binding of thrombin to protease-activated receptors, not protecting the integrity of endothelial barriers [133].

Dabigatran is chemically different and more hydrophilic than other NOACs, such as Rivaroxaban, even if both demonstrated the capability of inhibiting the induction of ROS production on an in vitro model of vascular endothelial damage using HUVEC stimulated with 25-hydroxycholesterol (25-OHC) [134]. Moreover, Dabigatran demonstrated a higher antioxidant activity than Rivaroxaban.

In circulating cells in vitro cultured, pre-treated with Dabigatran, then stimulated with thrombin or serum from healthy donors, the NOAC inhibited not only procoagulant effects in inflammatory conditions but also proinflammatory stimuli via reducing the expression/secretion of chemokines (namely, IL-8, CXCL1 and MCP-1), not affecting inflammatory cytokines and growth factors [135]. However, to the best of our knowledge, no miR targets of Dabigatran were validated, and a study about its inference on the expression of miR-27a-3p (predicted by miRDB engine [136]) to target coagulation molecules as Tissue factor and Tissue factor pathway inhibitors) suggested the absence of a functional interrelationship [137].

## 4. Digoxin

Digoxin, derived from the purple foxglove flower, called with brand names Digox or Lanoxin (Table 1), is classified as a cardiac glycoside with inotropic cardioactivity [138,139], approved by the FDA in 1954. Digoxin reversibly inhibits the Na-K ATPase enzyme [140] (Table 1). Digoxin leads to various beneficial effects at low doses, stimulates the parasympathetic nervous system via the vagus nerve, helping to decrease norepinephrine levels, thus supporting heart rate control [138,141,142]. Although Digoxin is not an anticoagulant, the in vitro increase of platelet aggregation following Digoxin was reported so far [143]. Carefulness in Digoxin administration is also suggested in patients with HF and AF, due to the increase of both endothelial and platelet activation in patients with non-valvular AF [144,145] and the negative outcomes observed when Digoxin was co-administered with vitamin K antagonists or NOACs l [146,147,148].

Toxicity, morbidity, and mortality without benefit are known for administration at doses > 1.0 ng/mL [149]. Digoxin is largely administered to patients with HF, and its bioavailability varies from 50 to 100% for oral administration (maximum bioavailability reported for oral gelatinized capsules) [150]. A metabolism study showed that Digoxin is approximately 70–80% absorbed in the first part of the small bowel [151]. Although the colon microbiota could transform the drug into pharmacologically inactive byproducts, the intestinal P-glycoprotein/ABCB1 may interfere with pharmacokinetics [152]. Unchanged Digoxin is prevalently excreted in the urine [153].

In neuronal models, Digoxin transcriptionally upregulated the miR-132, protecting rat primary neurons and iPSC-derived human neurons against insults [154]. Conversely, the miR-132 drives in vivo the pathological growth of cardiomyocytes in a murine model of left ventricular pressure overload [155], and was suggested as a therapeutic target in HF and cardiac diseases [156,157]. In fact, miR-132 inhibition partially rescued severe left ventricular (LV) hypertrophy, and reduced ejection fraction (EF) and cardiac dilatation in miR-212/132-TG mice 6 weeks old [158]. Analogously, an improvement of cardiac function following treatment with DR132L was reported in a pig model of HF post-myocardial infarction [159]. In humans, a randomized clinical trial phase 1b (NCT04045405) in 28 HFrEF patients tested safety, tolerance, and efficacy, and plasma pharmacokinetics of a first-in-class miR-132 inhibitor, namely DR132L (a specific antisense oligonucleotide), showing preliminary promising results at cardiac functional level [160]. These findings lead to the multicentric Phase 2 trial HF-REVERT, the first trial testing an antisense oligonucleotide-based microRNA inhibitor in human HF, which is currently enrolling patients with HF and preserved or moderately reduced ejection fraction (n = 280) randomized to two doses of DR132 or placebo in addition to standard therapy [159,161]. The bipolarity of Digoxin is highlighted by these studies: if on one side the drug regulates the cardiac rate, on the other, it could interfere with platelet response and cardiac function. The HF-REVERT trial, not excluding patients on Digoxin therapy, will possibly establish whether an interplay between Digoxin and pharmacological inhibition of miR-132 or platelet aggregation may exist (Supplementary Figure S6A) in human HF. At present, in vivo interference between DR132L and Digoxin cannot be excluded. Overall, these data will be relevant in the optics of including miR inhibitors in HF in a therapeutic approach and ultimately in GDMT.

In vivo treatment of RGS2−/− mice with Digoxin stabilized the Regulator of G protein signaling 2 (RGS2), a G(q)-specific GTPase-activating protein, implicated in hypertension and cardiovascular function [162]. In silico analysis shows the hsa-miR-4717-5p and hsa-miR-22-3p (biomarker of adverse events in HF) as strongly validated targets of RGS2 and a connection between hsa-miR-22-3p and miR-132 via SIRT1 (Supplementary Figure S6B). Although no direct functional relationship between these miRs and Digoxin is currently reported, in silico evidence relating at cardiac level the SIRT1 gene to Digoxin is available [163]. Moreover, a not FDA-approved Digoxin functional and structural analogous isolated from milkweed plants of the family Asclepiadoideae [164], namely Calotropin, is active over HF by modulating SIRT1/FOXD3/SERCA2a pathway [165], thus supporting the relevance of this pathway in the action mechanism of cardiac glycosides. This preliminary information, together with the notion that SIRT1 plays a role in thrombosis, fosters further studies to elucidate the possible relationships among RGS2, SIRT1, and miR-22 in patients with HF treated with Digoxin.

## 5. Sino-Atrial Node Current Inhibitor: Ivabradine

Ivabradine was approved by FDA in 2015 with the commercial name of Procoralan and Lancora, and by EMA in 2017 (Table 1).

Ivabradine belongs to the family of benzazepines and is a HCN channel blocker, selectively inhibiting the “funny” channel pacemaker current (I_f_) [166,167,168], which implicates particular care in administration to patients with a pacemaker. Ivabradine binds by entering and attaching to a site on the channel pore from the intracellular side and disrupts I_f_ current flow, thus prolonging diastolic depolarization, lowering heart rate [169,170].

Ivabradine is metabolized by oxidation in the gut and liver by cytochrome CYP3A enzyme, and concomitant use of CYP3A inhibitors is contraindicated because increasing the risk of fatal arrhythmias [168]. Metabolites are equally excreted in faeces and urine.

Ivabradine use aimed at reducing hospitalization of patients with severe HF and elevated sinus rhythm is indicated, sometimes as a synergistic combination with beta blockers [169,170]. However, some debate persists about its role in inducing AF, as well as on the appropriateness of prescribing Ivabradine to patients with paroxysmal AF [171], thus investigations on the Ivabradine action mechanisms are still ongoing. In the transgenic (TG) AF murine model with heart-specific overexpression of the (pro) renin receptor, Ivabradine significantly decreased the incidence of AF. In this model, the treatment with Ivabradine inhibits the upregulation of HCN2 and HCN4 protein expression in the atrial tissue, rescuing the loss of function and reversing the electrophysiological cardiac maladaptive remodeling [172]. Consistently, Ivabradine lowered blood pressure, improved cardiac remodeling and inflammation, and decreased renal damage in SHR hypertensive rats, through increasing renal HCN2 mRNA and reducing cardiac HCN4 mRNA [173].

Due to the different action mechanisms, the interference of Ivabradine with anticoagulant/antiplatelet drugs is currently not considered, and the producer declared no interference between Ivabradine and Warfarin (see Corlanor _prescribing information by Amgen Inc.; April 2015). However, the amelioration of cardiac rate in HF by Ivabradine was related in both humans and mice to the upregulation of miR-133, which is also targeted by Warfarin [170,174] (Figure 3). This association of miR-133 with both Ivabradine and Warfarin dose efficacy may be relevant. It cannot be excluded a priori that an increase of miR-133 following Ivabradine affects VROCK1, thus amplifying or antagonizing the Warfarin effect. For completeness, it is worth mentioning that another miR was associated to downregulation of the pacemaker ion channel HCN4 in mice following Ivabradine, namely miR-370-3p [175], but this miR is not interconnected with miR-133 (as shown also by silico analysis by miRTargetLink 2.0 [44], Supplementary Figure S7) or the coagulation. Moreover, preliminary data on the absence of reciprocal interference between Ivabradine and Clopidogrel have been reported [176]. The paucity of information on the molecular mechanism associated with Ivabradine action paves the way to deep investigations for establishing whether the functional connection of Ivabradine with miR-133 leads to an inference of Ivabradine in coagulation-related outcomes and drug resistance.

## 6. Conclusions

In HF, rising incidence and clinical complexity are paralleled by not always optimized GDMT and adverse events due to drug resistance or therapeutic failures. The different structure, metabolism and targets of the prescribed drugs only partially explain the existence of broad differences in patient responsivity.

In this panorama, the integration of clinical pharmacology with multi-omics approaches, the identification of genetic polymorphisms and epigenetic mechanisms, particularly of the regulation mediated by specific miRs, will provide molecular insights into the fine-tuned mechanism at the base of the variability in individual responses to anticoagulant drugs and of drug resistance in HF. Identification of miRs changing upon drug administration or paralleling patient response could provide drug-related biomarkers useful in calibrating therapy dose or monitoring efficacy over time in patients where hemostasis maintenance is crucial. We may speculate that a deep knowledge of functional relationships between miRs and anticoagulant drugs will also allow indirect intervention on drug efficacy or resistance via modulating circulating or tissue levels of specific miRs.

Although in its infancy, the literature on the relationship between non-coding RNA with widely used anticoagulant/antiplatelet drugs (e.g., Clopidogrel, Aspirin, Warfarin, and NOACs) and with Digoxin and Ivabradine is already enhancing the knowledge on the molecular mechanism associated with HF treatment. Up to now, several miRs (e.g., miR-133, miR-26a/b, and miR-137) were specifically associated to platelet reactivity, coagulation pathways, and cardiovascular remodeling upon anticoagulant administration. It is worth highlighting that the family of miR-26 was reported in relationship to Clopidogrel (miR-26a) and Aspirin (miR-26b), thus could represent a candidate for pharmacological studies. The inhibition of VROK1 by miR-133 and miR-137 in Warfarin- or Ivabradine-dependent or independent ways, also deserves further investigation aimed at understanding if the two drugs synergize in VROCK1 inhibition, if miR-137 might contribute to the enhancement of Warfarin efficacy, and how myocardial alteration in HF stems from miR-137 deregulation. Although not focused on anticoagulant drugs, the HF-REVERT trial on the miR-132 antagomiR, namely DR132, is the first example in the field of translating into clinical cardiac practice—and specifically applying to HF—the results from studies on miRs, thus supporting the translational potential of miR modulation.

Overall, the studies focusing on the interaction between miRs and drugs represent an innovative branch in HF research. Therefore, due to rapid technological advancements, the miR-based modulatory therapies will be feasible in a not-so-distant future. The most striking example is provided by the rapidity of development of RNA-based vaccines against COVID-19. Notably, the RNA-based vaccines had the limitation of requiring administration by injection. However, a recent article introduced the possibility of oral delivery of complexed mRNA via encapsulation in a capsule device with a coating that dissolves at intestinal but not gastric pH. This formulation was successfully tested in mice, rats, and swine [177]. Encapsulation of different RNAs, including miRs, and translation to humans for the widest application, represent the next steps. The optimization of standardized protocols for quantifying circulating/tissue miRs, alongside the development of miR inhibitors or enhancers together with that of advanced targeting delivery carriers/systems (ensuring tissue specificity and molecular stability), will be critical for clinical translation of the miR-based diagnostics and therapeutics.

An integrative strategy, balancing the doses of anticoagulant drugs and oral antagomiRs, will, in perspective, allow refining patient stratification, optimizing more personalized therapeutic interventions, decreasing drug resistance, ultimately facilitating clinical activity while improving the clinical outcomes of patients with HF.

## Figures and Tables

**Table 1:** Useful links for the studied drugs to pharmacological and action mechanism data on websites (all accessed on 21 August 2025).

Drug	Pharmaceutical Data *	Basic Information by FDA	Basic Information by EMA	Small Molecule Pathway Database Action Pathway **
**Clopidogrel**	https://go.drugbank.com/drugs/DB00758	https://www.accessdata.fda.gov/drugsatfda_docs/label/2016/020839s062s064lbl.pdf	https://www.ema.europa.eu/en/medicines/human/EPAR/plavix	https://smpdb.ca/search?q=clopidogrel
**Ticagrelor**	https://go.drugbank.com/drugs/DB08816	https://www.accessdata.fda.gov/drugsatfda_docs/label/2016/022433s020lbl.pdf	https://www.ema.europa.eu/en/medicines/human/EPAR/brilique#overview	unavailable
**Aspirin**	https://go.drugbank.com/drugs/DB00945	https://www.fda.gov/search?s=aspirin&sort_bef_combine=rel_DESC	https://www.ema.europa.eu/en/documents/psusa/acetylsalicylic-acid-list-nationally-authorised-medicinal-products-psusa00000039201602_en.pdf	https://smpdb.ca/view/SMP0000083?highlight[compounds][]=DB00945&highlight[proteins][]=DB00945
**Warfarin**	https://go.drugbank.com/drugs/DB00682	https://www.accessdata.fda.gov/drugsatfda_docs/label/2011/009218s107lbl.pdf	https://catalogues.ema.europa.eu/search?search_api_fulltext=WARFARIN&conjunction=OR	https://smpdb.ca/search?q=warfarin
**Apixaban**	https://go.drugbank.com/drugs/DB06605#label-reference	https://www.accessdata.fda.gov/drugsatfda_docs/label/2016/202155s012lbl.pdf	https://www.ema.europa.eu/en/medicines/human/EPAR/eliquis	https://smpdb.ca/view/SMP0126437
**Rivaroxaban**	https://go.drugbank.com/drugs/DB06228	https://www.accessdata.fda.gov/drugsatfda_docs/label/2021/215859s000lbl.pdf	https://www.ema.europa.eu/en/medicines/human/EPAR/xarelto#overview	https://smpdb.ca/search?q=Rivaroxaban
**Edoxaban**	https://go.drugbank.com/drugs/DB09075	https://www.accessdata.fda.gov/drugsatfda_docs/label/2015/206316lbl.pdf	https://www.ema.europa.eu/en/medicines/human/EPAR/lixiana	https://smpdb.ca/view/SMP0126436
**Dabigatran etexilate**	https://go.drugbank.com/drugs/DB06695	https://www.accessdata.fda.gov/drugsatfda_docs/label/2024/022512s047lbl.pdf	https://www.ema.europa.eu/en/medicines/human/EPAR/pradaxa	https://smpdb.ca/search?q=Dabigatran+etexilate
**Digoxin**	https://go.drugbank.com/drugs/DB00390	https://verification.fda.gov.ph/files/DR-262_PI_01.pdf	unavailable	https://smpdb.ca/search?q=digoxin
**Ivabradine**	https://go.drugbank.com/drugs/DB09083	https://www.accessdata.fda.gov/drugsatfda_docs/label/2015/206143orig1s000lbl.pdf	https://www.ema.europa.eu/en/medicines/human/EPAR/ivabradine-anpharm	unavailable

Table Legend: * **DrugBank** is an online comprehensive repository of drugs, drug targets, and drug actions providing essential descriptive information for chemical and pharmacological compounds (i.e., Identification, Pharmacology, Interactions, Products, Categories, Chemical Identifiers, References, Clinical Trials, Pharmacoeconomics, Properties, Spectra, Targets, Enzymes, Carriers). ** **Small Molecule Pathway Database** (SMPDB) is an interactive, visual database with >30,000 pathways for small molecules (including drugs) in humans. It shows both action and metabolism pathways, a related description, and references.

## Data Availability

Not applicable.

## Supplementary Materials

The following supporting information can be downloaded at: https://www.mdpi.com/article/10.3390/biom15101411/s1, **Supplementary Figure S1**: miR-19b-1-5p target network. **Supplementary Figure S2**: miR-26b-3p target network. **Supplementary Figure S3**: miR-133 target networks. **Supplementary Figure S4**: miR-483-5p target network. **Supplementary Figure S5**: ABCB1 miR targets. **Supplementary Figure S6**: Putative role of Digoxin in platelet aggregation, and target networks. **Supplementary Figure S7**: Target networks.
