# Supplementary figures and images for: Anticoagulation Therapies and microRNAs in Heart Failure
Source: Biomolecules. 2025 Oct 3;15(10):1411. doi: 10.3390/biom15101411 (PMC12564667; doi:10.3390/biom15101411)

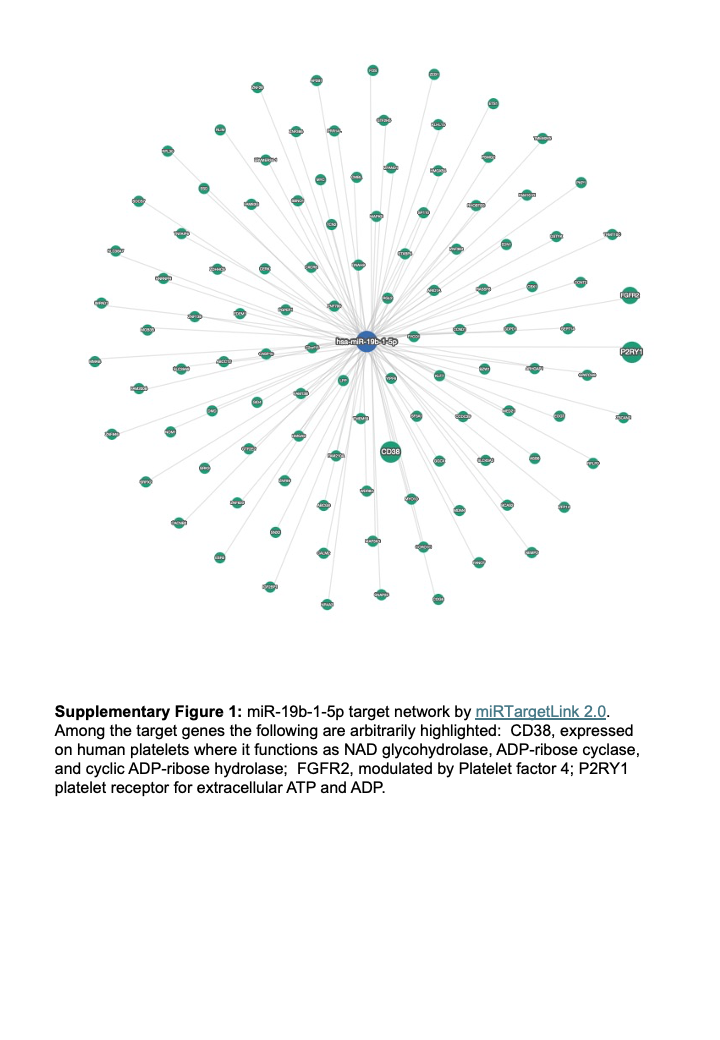

Supplement: Supplementary file 1 [file biomolecules-15-01411-s001.zip › Figure S1.tiff]

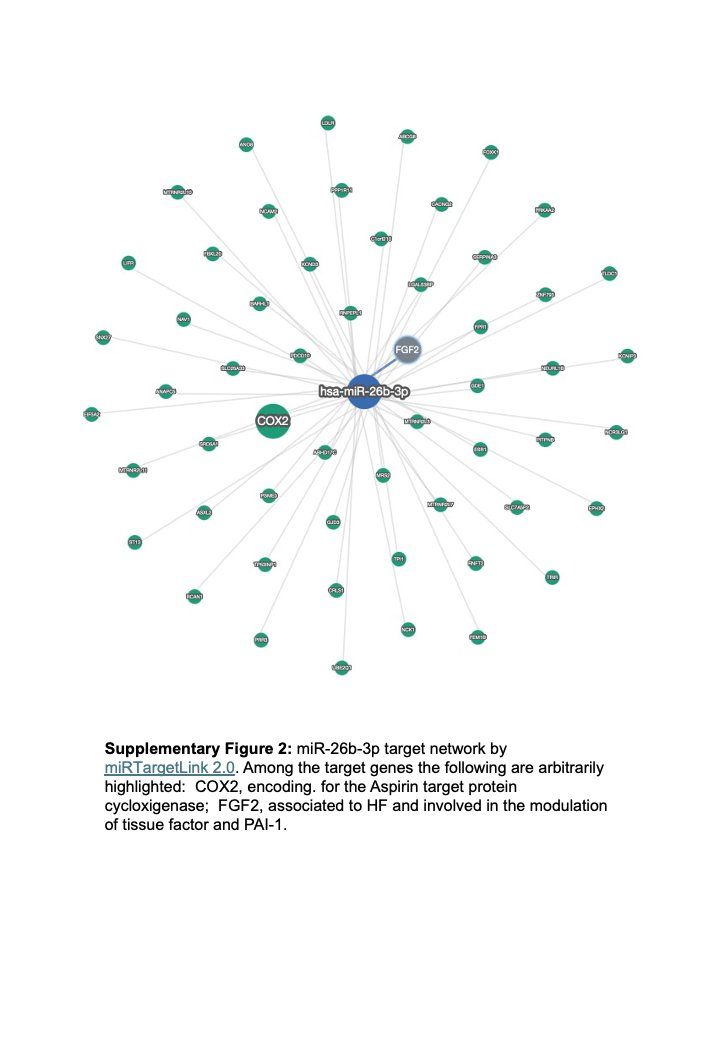

Supplement: Supplementary file 1 [file biomolecules-15-01411-s001.zip › Figure S2.tiff]

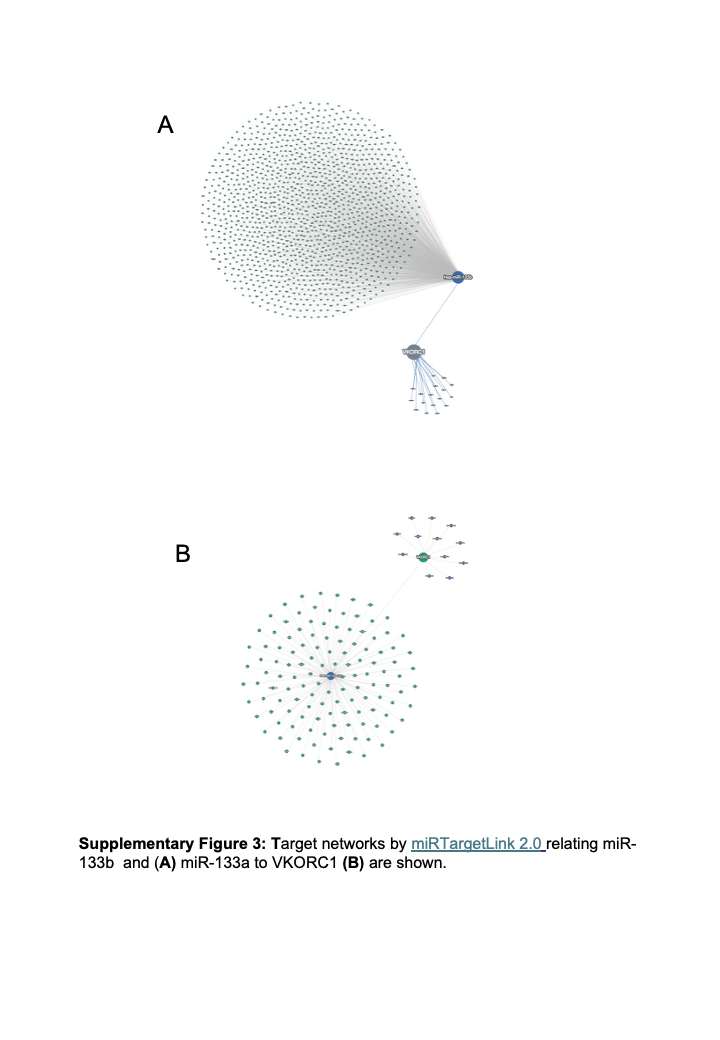

Supplement: Supplementary file 1 [file biomolecules-15-01411-s001.zip › Figure S3.tiff]

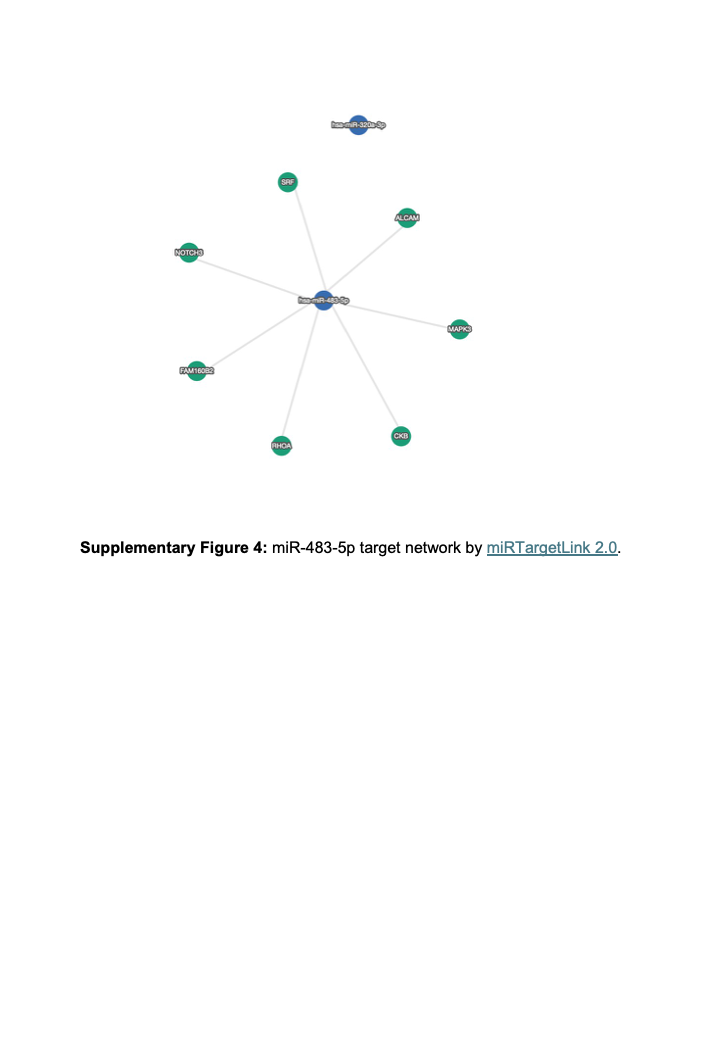

Supplement: Supplementary file 1 [file biomolecules-15-01411-s001.zip › Figure S4.tiff]

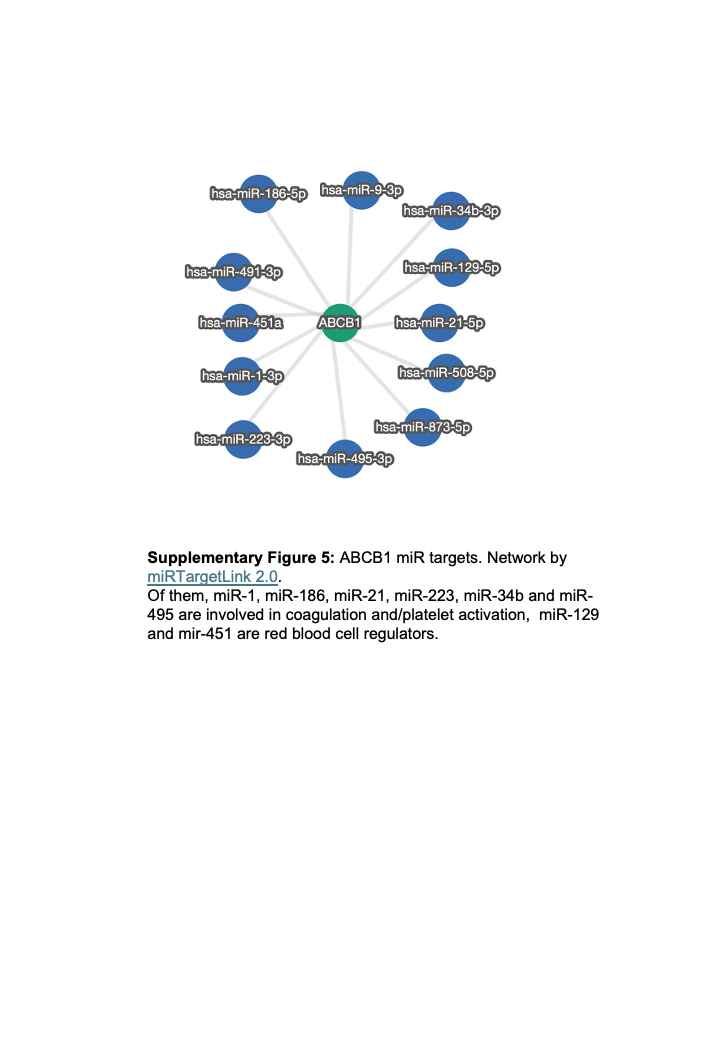

Supplement: Supplementary file 1 [file biomolecules-15-01411-s001.zip › Figure S5.tiff]

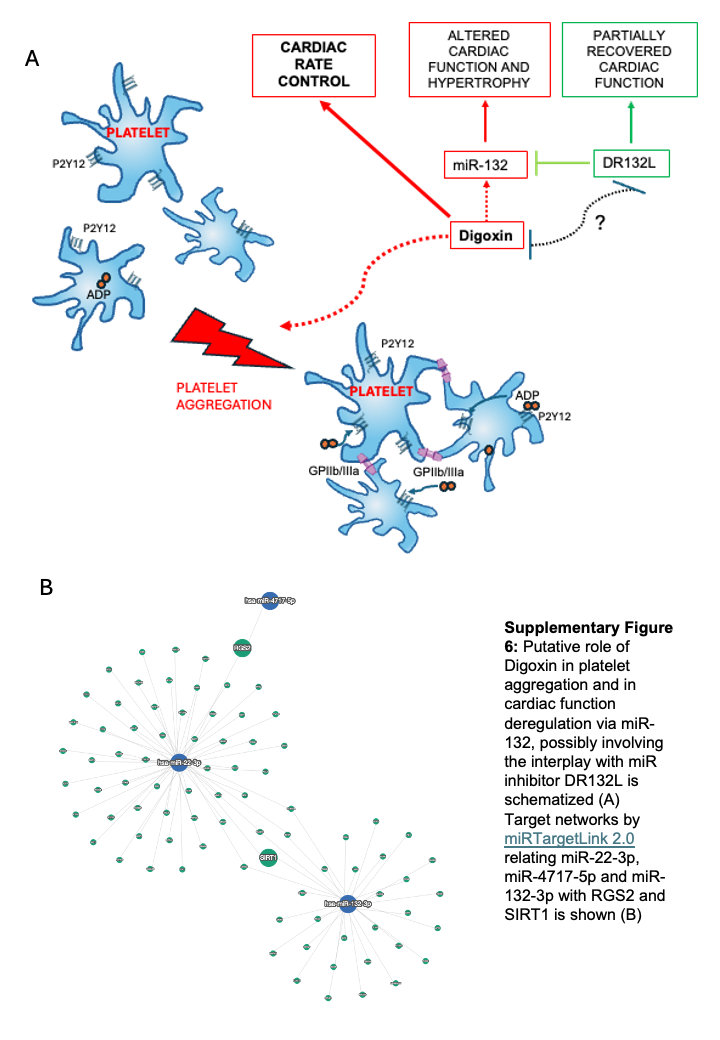

Supplement: Supplementary file 1 [file biomolecules-15-01411-s001.zip › Figure S6.tiff]

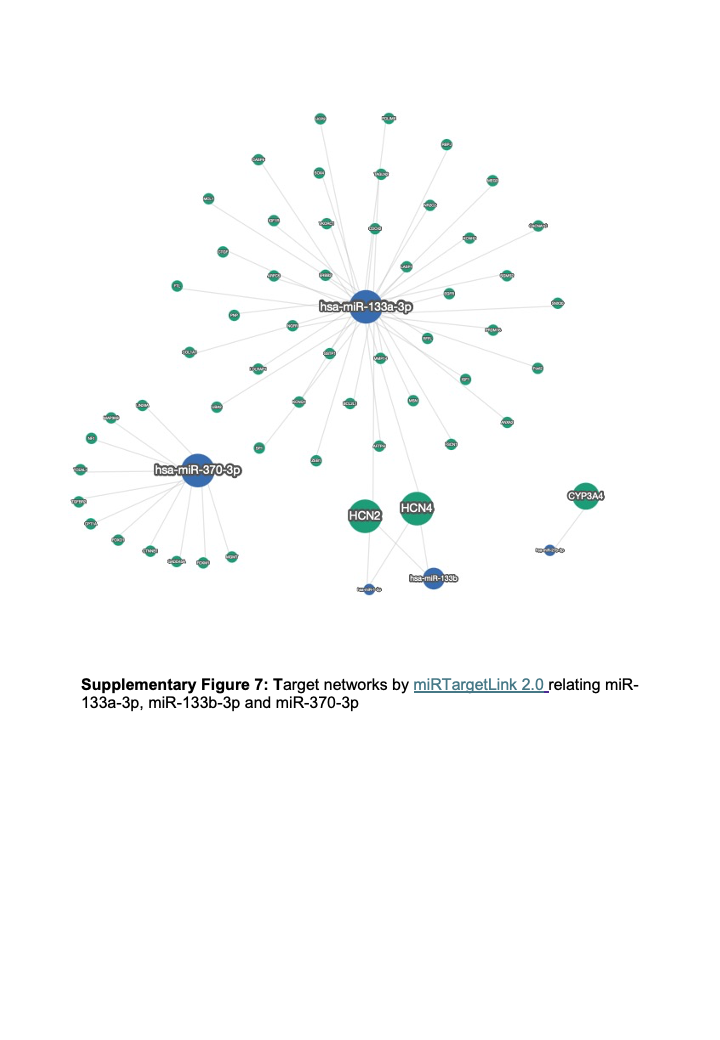

Supplement: Supplementary file 1 [file biomolecules-15-01411-s001.zip › Figure S7.tiff]
